# Klinischer Einsatz von Medizinstudierenden als personelle Überlaufkapazität in einer Notlage am Beispiel der Covid-19-Pandemie: Qualitative Auswertung der ESCAPE-Studie

**DOI:** 10.1007/s00103-026-04236-4

**Published:** 2026-05-04

**Authors:** Lucien Torlot, Johanna Huber, Carla Nau, Franziska Jahns, Bernhard Zwißler, Martin R. Fischer, Ines Schroeder, PREPARED Study Group, Claudia Apel, Marc Bodenstein, Iris Chaberny, Enrico Dähnert, Kristin Engelhard, Roland Francis, Thea Koch, Susanne Kolbe-Busch, Kerstin Lamers, Thomas von Lengerke, Muriell Madi, Brigitte Malien, Mario Menk, Maria Reden, Tiffany Schaumburg, Sasa Sopka, Sebastian Stehr, Astrid Stephan, Ivonne Tomsic, Irhad Trozic, Rojda Ülgüt, Steffen Weber-Carsten, Kathrin Zednik

**Affiliations:** 1 Klinik für Anaesthesiologie, LMU Klinikum, LMU München, Marchioninistr. 15, 81377 München, Deutschland; 2 Institut für Didaktik und Ausbildungsforschung in der Medizin, LMU Klinikum, LMU München, München, Deutschland; 3 Klinik für Anästhesiologie und Intensivmedizin, Universitätsklinikum Schleswig-Holstein, Campus Lübeck, Lübeck, Deutschland

**Keywords:** Notfall-Bereitschaft, Surge Capacity, Krankenhäuser, Belegschaft, Freiwillige Krankenhausmitarbeitende, Emergency preparedness, Surge capacity, Hospitals, Workforce, Hospital volunteers

## Abstract

**Hintergrund:**

Kliniken der Akutversorgung sollten eine personelle Überlaufkapazität (Surge Capacity) vorsehen, um auf medizinische Notlagen vorbereitet zu sein. Da Medizinstudierende eine mögliche Ressource darstellen, wurde untersucht, ob ihr geplanter Einsatz erstrebenswert ist und unter welchen Bedingungen dieser stattfinden könnte.

**Methoden:**

In 2 retrospektiven, multizentrischen Querschnittsstudien wurden im Jahr 2023 Medizinstudierende an 20 deutschen Fakultäten sowie Leitungskräfte aus kritischen Bereichen der Patientenversorgung an 2 Universitätsklinika befragt. Gegenstand der Befragung waren studentische Einsätze während der COVID-19-Pandemie. Zudem wurden die Bereitschaft für zukünftige studentische Einsätze und die dafür notwendigen Rahmenbedingungen ermittelt.

**Ergebnisse:**

Leitungskräfte (*n* = 72) und Studierende (*n* = 1249) bekundeten eine deutliche Bereitschaft zur Zusammenarbeit in einer zukünftigen Notlage (jeweils 81,9 % und 90,8 % Zustimmung) durch Übertragung diagnostischer, assistierend ärztlicher, pflegerischer oder logistischer Tätigkeiten. Aus studentischer Sicht sollten dafür eine adäquate Entlohnung, klare Regelungen in Bezug auf akademische Pflichten und eine suffiziente Einarbeitung etabliert werden. Die Leitungskräfte forderten die fachliche Befähigung der Hilfskräfte. Als limitierenden Faktor wurden vor allem die akademischen Verpflichtungen der Studierenden wahrgenommen.

**Schlussfolgerungen:**

Diese Arbeit stellt die größte uns bekannte Erhebung zu einer studentischen Überlaufkapazität dar, die in einer erneuten Notlage als ein sinnvolles und akzeptiertes Konzept erscheint. Diese Ergebnisse unterstützen die Erarbeitung von Tätigkeitsbeschreibungen für Hilfskräfte, ihre präemptive Schulung und den klaren Umgang mit universitären Pflichten.

**Zusatzmaterial online:**

Zusätzliche Informationen sind in der Online-Version dieses Artikels (10.1007/s00103-026-04236-4) enthalten.

## Abstract (en)

**Background:**

Hospitals should plan a staffing surge capacity to prepare for a new pandemic or disaster. As medical students are a possible surge capacity resource, this study investigated whether including students in surge capacity plans is desirable and under which conditions it is feasible.

**Methods:**

We performed two retrospective multicentre observational trials in 2023 by enrolling medical students from 20 German faculties and leadership from critical areas of inpatient care at two university hospitals. We investigated the integration of medical students in the COVID-19 effort, assessed the willingness to include students in future preparedness plans, and explored possible requirements for such a deployment.

**Results:**

Both leadership staff (*n* = 72) and students (*n* = 1249) displayed a clear willingness for cooperation in the context of preparedness plans (81.9% and 90.8% approval respectively). Most agreed on delegating diagnostic, medical assistance, nursing and logistical tasks to the surge capacity help force. Students addressed financial compensation, clearance of academic obligations and sufficient training as prerequisites for their deployment. Leadership staff also emphasized the need for adequate training. Both parties cited students’ academic obligations amongst the main limiting factors.

**Conclusion:**

Both surveys display a high acceptance for including students into the surge capacity workforce. These results support the need for predefined roles for surge capacity helpers and emphasize the necessity of pre-emptive preparedness training and the clarification of academic obligations ahead of time.

## Hintergrund

Während der COVID-19-Pandemie bestand ein erhöhter Personalbedarf zur Sicherstellung der medizinischen Grundversorgung und zur Kompensation der erheblichen krankheits- und isolationsbedingten Personalausfälle. Um ausreichend Personal für die medizinische Grundversorgung und die Behandlung von COVID-19-Patienten sicherzustellen, kam es in Deutschland unter anderem zur Reduktion von elektiven Behandlungen und Reallokation von Personal aus nichtkritischen Bereichen sowie zur Rekrutierung von niedergelassenen, pensionierten und ausländischen Fachkräften [1, 2]. Die Weltgesundheitsorganisation (WHO) empfiehlt seit 2011 den zusätzlichen Einsatz von freiwilligen Helfenden in medizinischen Notlagen [3, 4]. Dies wurde während der COVID-19-Pandemie in Deutschland und weltweit in unterschiedlichem Maße für die Unterstützung der Patientenversorgung und der öffentlichen Gesundheitsdienste umgesetzt. In den meisten europäischen Ländern wurden dabei präferenziell Medizinstudierende in die Pandemiebewältigung eingebunden. Dabei leisteten sie zum Teil einen signifikanten Beitrag zur Pandemiebewältigung, beispielsweise durch Unterstützung des Öffentlichen Gesundheitsdienstes und ihren Einsatz in der Patientenversorgung, unter anderem in der COVID-19-Testung [5, 6]. In Ländern wie Deutschland, Griechenland und den USA unterstützten zusätzlich ehrenamtliche Helfende aus Hilfsorganisationen Abläufe in Kliniken, Impf- und Testzentren. Zuletzt griffen einige Länder auf etablierte „medizinische Reservekapazitäten“ zurück, beispielsweise das US-amerikanische Medical Reserve Corps. Diese vermittelten neben Fachkräften, die nicht hauptberuflich im Gesundheitswesen tätig waren, auch Ehrenamtliche mit wenig medizinischen Kenntnissen [5, 6].

Welche Konzepte hier in Deutschland auch für zukünftige medizinische Notlagen geeignet sind, wurde kürzlich in einer Übersichtsarbeit der Autor:innen zusammenfasst [6]. Hierbei wurden – analog der COVID-19-Pandemie – unter anderem Helfende des deutschen Katastrophenschutzes sowie Studierende der Gesundheitsberufe als Personalquellen für zukünftige Preparedness-Pläne identifiziert.

Der Einsatz von Medizinstudierenden bietet dabei spezifische Vorteile: Sie verfügen über praktische Vorkenntnisse in der klinischen Evaluation von Patienten und in grundlegenden pflegerischen und diagnostischen Tätigkeiten. Sie sind zusätzlich mit den Strukturen und den Behandlungsteams ihrer Lehrkrankenhäuser vertraut, besitzen eine starke intrinsische Motivation, in Notlagen zu helfen, und können aus einem solchen Einsatz didaktischen Nutzen ziehen. Trotz bestehender Vorkenntnisse müssen sie in aller Regel geschult und in Arbeitsbereiche und -abläufe eingewiesen werden und ihre akademischen Verpflichtungen können mit ihrer Verfügbarkeit als Hilfskräfte kollidieren.

Der Einsatz von Medizinstudierenden in der COVID-19-Pandemie war in der Regel spontan und kurzfristig geplant. In der vorliegenden Studie wurde untersucht, ob der geplante und strukturierte Einsatz von Medizinstudierenden in Preparedness-Pläne wünschenswert ist und unter welchen Voraussetzungen dieser stattfinden könnte. Folgende Fragestellungen sollten beantwortet werden:

- Wie waren Studierende in der Pandemie involviert und haben sie ihren Einsatz als hilfreich empfunden?
- Welche Vor- und Nachteile sowie Limitationen entstehen den Studierenden aus einem solchen Einsatz?
- Sind Studierende und klinische Leitungskräfte an einem zukünftigen, geplanten Einsatz von Studierenden interessiert und welche Rahmenbedingungen müssen dafür erfüllt werden?

## Methoden

Die ESCAPE-Studie (Evaluating Surge Capacity And PrEparedness) entstand im Kontext des PREPARED-Projektes (PREpardness and PAndemic REsponse in Deutschland) des Netzwerks Universitätsmedizin im Arbeitspaket AP 10 (Personalmanagement; [7]).

### Studiendesign

Es wurde unter Medizinstudierenden und klinisch tätigen Leitungskräften jeweils eine multizentrische Querschnittsstudie nach den STROBE-Kriterien (Strengthening the Reporting of Observational Studies in Epidemiology) durchgeführt [8]. Beide Umfragen wurden in einem mehrstufigen Prozess in einer Expertengruppe bestehend aus 2 leitenden Intensivmedizinern (Erfahrung im klinischen Management und in der Personalplanung während der Pandemie), einem Studiendekan und einer Gesundheitswissenschaftlerin (Erfahrung im Umfragedesign und Datenauswertung) erstellt. Beide Umfragen (Studierenden- und Leitungskräfteumfrage) beinhalteten 2 Teile mit jeweils 17 und 11 Fragen:

1. demografische Angaben und bisherige Erfahrungen als sogenannte Pandemie-Helfende während der COVID-19-Pandemie;
2. Bereitschaft zur Mitarbeit und Einschätzung des Nutzens einer studentischen Überlaufkapazität im Rahmen einer zukünftigen medizinischen Notlage; Definition potenziell delegierbarer Tätigkeiten; notwendige Rahmenbedingungen für den Einsatz; Vor- und Nachteile eines solchen Einsatzes.

Die Umfragen beinhalteten unter anderem Multiple-Choice-Fragen, offene Fragen sowie Fragen zur Zustimmung, die auf einer 6‑stufigen Likert-Skala beantwortet wurden. Bei der Antwortmöglichkeit „andere“ oder bei fehlender Zustimmung wurden Freitextantworten ermöglicht. Die Umfragen wurden mit der Befragungssoftware evasys V9.1 durchgeführt (evasys GmbH, Lüneburg, Deutschland).

Beide Umfragen wurden durch 3 selektierte Leitungskräfte und 2 Studierende pilotiert und iterativ verbessert.

Die Umfragen sind in der deutschen Fassung im Onlinematerial 1 verfügbar.

### Rekrutierung

Die Studiendekanate der 38 medizinischen Fakultäten in Deutschland erhielten eine E‑Mail-Einladung zur Teilnahme an der Studierendenumfrage. Nach Zustimmung seitens der Dekanate wurde die Verteilung der Umfrage mittels E‑Mail-Verteiler und/oder digitaler Schwarzer Bretter realisiert. Die meisten Fakultäten verschickten eine Erinnerungsnotiz an die Teilnehmenden. Die Befragung fand zwischen dem 16.10. und 27.11.2023 statt, war freiwillig und anonym.

Für die Leitungskräfteumfrage wurden in einem iterativen Prozess die kritischen Bereiche der klinischen Versorgung während einer Pandemie definiert. Die Leitungskräfte möglichst aller Berufsgruppen (ärztlich, pflegerisch, technische Assistenz) dieser Bereiche am Klinikum der Universität München (LMU Klinikum) und am Universitätsklinikum Schleswig-Holstein (UKSH) in Lübeck wurden per E‑Mail zur freiwilligen und anonymen Teilnahme eingeladen. Aufgrund der strengen Datenschutzbestimmungen konnte die Leitungskräfteumfrage an weniger Standorten als geplant durchgeführt werden. Die Umfrage wurde an den jeweiligen Standorten durch den Personalrat bewilligt.

### Analyse

Die statistischen Analysen wurden mit SPSS Statistics 29 (IBM, Armonk, New York) durchgeführt. Für demografische und quantitative Daten wurden deskriptive Statistiken (relative Häufigkeit, Mittelwert und Standardabweichung) ermittelt. Die Normalverteilung wurde anhand des Shapiro-Wilk-Tests überprüft (*p* < 0,001). Zur Berechnung von Gruppenunterschieden im Hinblick auf das Geschlecht, das Ausbildungsjahr und die Berufsgruppe wurden für kategoriale Daten der Pearsons-Chi-Quadrat-Test und für nichtparametrische, intervallskalierte Daten der Kruskal-Wallis-Test angewendet. Das Signifikanzniveau wurde auf 0,05 festgelegt. Zur Beurteilung des Effekts signifikanter Unterschiede wurde für kategoriale Variablen Cramérs V, für nicht normalverteilte intervallskalierte Variablen die Effektgröße *r* berechnet. Cohen klassifiziert die Effektgrößen V und *r* wie folgt: > 0,1 kleiner Effekt, > 0,3 moderater Effekt, > 0,5 großer Effekt [9].

Qualitative Daten wurden mittels der inhaltlich strukturierenden qualitativen Inhaltsanalyse nach Kuckartz mithilfe eines deduktiv-induktiv entwickelten Kategoriensystems ausgewertet [10]. Da es in diesem Forschungsfeld nur begrenzte qualitative Datensätze gibt, wurde die Kategorienentwicklung deduktiv anhand weniger publizierter Ergebnisse abgeleitet und induktiv ergänzt und erweitert [6, 11]. Es wurde auf die Bildung von Subkategorien aufgrund der eingeschränkten Informationsdichte in den einzelnen Datensegmenten verzichtet. Jede Freitextantwort wurde als Datensegment behandelt und nach den Kategorien im Codierleitfaden codiert (Onlinematerial 2). Die Freitextantworten beider Umfragen wurden vollständig durch LT codiert. Zur Überprüfung der Reliabilität des Codierleitfadens erfolgte bei 20 % der Freitext-Codierungen der Studierendenumfrage und bei 100 % der Freitext-Codierungen der Leitungskräfteumfrage eine zweite unabhängige Kodierung durch JH bzw. IS. Diskrepanzen zwischen den Codierungen wurden besprochen und durch Anpassung des Codierleitfadens beseitigt. Im Rahmen dessen wurde deutlich, dass einzelne Freitextantworten aufgrund ihrer inhaltlichen Mehrdeutigkeit von 2 unabhängigen Codierenden als „nicht codierbar“ bewertet wurden. In einigen Fällen konnten die betreffenden Textstellen als „nicht zutreffend“ für die gestellte Frage, aber als relevant für eine andere Frage der Umfrage angesehen und dementsprechend als Antwort der anderen Frage zugeordnet und codiert werden. Der angepasste Codierleitfaden wurde dann erneut angewendet. Die Reliabilität der Codierung wurde mittels Cohens Kappa ermittelt und ergab für die Studierendenumfrage einen Wert von 0,942 und für die Leitungskräfteumfrage einen Wert von 0,804. Die Häufigkeit der einzelnen Kategorien in den Umfragen wurde dann ermittelt.

## Ergebnisse

### Teilnehmende

Studierende von 20 der 38 deutschen medizinischen Fakultäten (52,6 %) nahmen an der Umfrage teil (Onlinematerial 3). Etwa 45.500 Studierende waren zu diesem Zeitpunkt an den teilnehmenden Fakultäten immatrikuliert. 1249 Studierende nahmen an der Umfrage teil, was einer konservativ geschätzten Teilnahmequote von mindestens 3 % entsprach. Alle 6 Studienjahre waren repräsentiert, 67,0 % der Teilnehmenden identifizierten sich als weiblich (*n* = 833) und das mediane Alter betrug 24 Jahre (Tab. 1).

**Tab. 1:** Soziodemografische und ausbildungsbezogene Merkmale der Studierenden sowie berufliche Merkmale der Leitungskräfte

*Studierende*		
Alter (Jahre)	Median (SD)	
	24 (3,6)	
Studienjahr	Anzahl (Anteil in %)	
	1	90 (7,2)
	2	146 (11,7)
	3	255 (20,4)
	4	285 (23,0)
	5	329 (26,5)
	6	103 (8,3)
	≥ 7	36 (2,9)
Geschlecht	Anzahl (Anteil in %)	
	Weiblich	833 (67,0)
	Männlich	395 (31,8)
	Divers	3 (0,2)
	k. A.	10 (1,0)
*Leitungskräfte*		
Berufsgruppe	Anzahl (Anteil in %)	
	Pflegende	26 (36,0)
	Ärzteschaft	35 (49,0)
	Andere	11 (15,0)
Abteilung/Arbeitsbereich	Anzahl (Anteil in %)	
	ITS/IMC	36 (50,0)
	Notaufnahme	2 (2,7)
	Normalstation	14 (19,4)
	Hygiene	2 (2,7)
	Laboratoriumsmedizin	3 (4,2)
	Sonstiger klinischer Bereich	9 (12,5)
	Sonstiger nichtklin. Bereich	5 (6,9)
	k. A.	2 (2,7)

*k.* *A.* keine Angabe; *ITS* Intensivstation; *IMC* Intermediate Care Unit; *SD* Standardabweichung

Die Expertenumfrage wurde an circa 220 medizinische Leitungskräfte verschickt und von 72 Teilnehmenden ausgefüllt (33,0 %). Die Mehrzahl waren ärztliche Mitarbeitende (49,0 %; *n* = 35) sowie Gesundheits- u. Krankenpflegekräfte (36,0 %; *n* = 26). Die Teilnehmenden waren überwiegend in der klinischen Patientenversorgung tätig (83,3 %; *n* = 60; Tab. 1).

### Studentische Überlaufkapazitäten während der COVID-19-Pandemie

In der Umfrage wurden die Erfahrungen studentischer Hilfskräfte während der Pandemie erhoben. Unter allen studentischen Teilnehmern gaben 42,0 % (*n* = 522) an, zur Pandemiebewältigung beigetragen zu haben. Von diesen Studierenden gaben 53,8 % (*n* = 281) an, klinische Tätigkeiten und 46,1 % (*n* = 241) nichtklinische Tätigkeiten durchgeführt zu haben. Signifikante Gruppenunterschiede zeigten sich hinsichtlich des Geschlechts und des Ausbildungsjahres. Männliche Studierende oder Studierende höherer Semester waren signifikant häufiger als studentische Hilfskräfte tätig als weibliche Studierende oder Studierende niedrigerer Semester (Geschlecht: Pearson-Chi-Quadrat = 9,34; *p* = 0,007; Cramérs V = 0,089; Ausbildungsjahr: Pearson-Chi-Quadrat = 38,02; *p* < 0,001; Cramérs V = 0,175). Beide Ergebnisse weisen eine geringe Effektstärke auf.

**Abb. 1:**
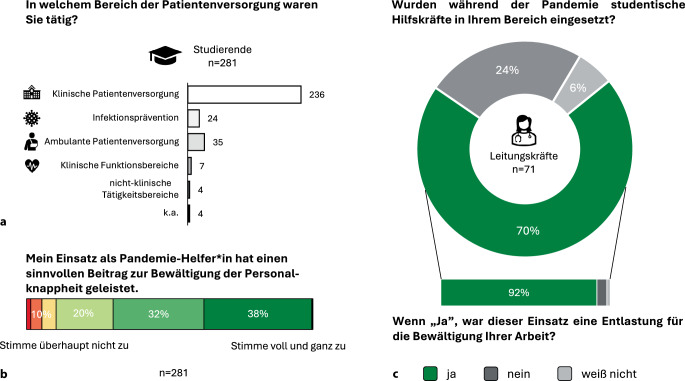
Einsatz von studentischen Überlaufkapazitäten während der COVID-19-Pandemie. **a** Anzahl der studentischen Pandemie-Helfenden pro Tätigkeitsbereich in der Patientenversorgung. Einige Studierende waren in mehreren Bereichen tätig. Codierleitfaden der Tätigkeitsbereiche im Onlinematerial 2. **b** Wahrgenommene Sinnhaftigkeit im Rahmen der Einsätze aller klinisch tätigen studentischen Pandemie-Helfenden (6-Punkte-Likert-Skala: „stimme überhaupt nicht zu“ bis „stimme voll und ganz zu“; k. A.: *n* = 1). **c** Anteil an Leitungskräften mit Einsatz von Pandemie-Helfenden im eigenen Arbeitsbereich sowie wahrgenommene Entlastung durch die Hilfskräfte. k. a.: keine Angabe

Studierende, welche in der Pandemie in der Patientenversorgung tätig waren (*n* = 281), gaben an, zu 85,2 % im stationären Bereich (*n* = 236), zu 12,6 % im ambulanten Bereich (*n* = 35) und zu 8,7 % im Bereich der klinischen Infektionsprävention (*n* = 24) tätig gewesen zu sein (Abb. 1a).

Von diesen Studierenden stimmten 89,0 % (*n* = 251) der Aussage: „Ich hatte das Gefühl, dass ich mit meinem Einsatz als Pandemie-Helfer:in einen sinnvollen Beitrag zur Bewältigung der Personalknappheit während der Pandemie leisten konnte“, zu oder voll und ganz zu (Mittelwert: M = 5,20; Standardabweichung: SD = 0,78; Abb. 1b). Im Hinblick auf das Geschlecht der Teilnehmenden konnten keine signifikanten Unterschiede festgestellt werden (Mann-Whitney-U-Test, *p* = 0,082). Studierende aus niedrigeren Semestern schätzten ihren Einsatz als hilfreicher als ihre erfahreneren Kommilitonen ein (*p* = 0,013, *r* = 0,15). Wenige empfanden ihre Arbeit als wenig hilfreich (10,0 %; *n* = 29) und begründeten dies mit dem falschen Einsatz personeller Ressourcen (*n* = 13), z. B. durch Überfluten eines Arbeitsbereichs mit Hilfskräften und mit einer mangelnden fachlichen Sicherstellung ihrer Einsatzfähigkeit (*n* = 5).

Unter den Leitungskräften meldeten 70,4 % (*n* = 50), Unterstützung durch Studierende erhalten zu haben. Davon gaben 92,0 % (*n* = 46) an, dass diese Hilfskräfte zur Bewältigung der pandemiebedingten Arbeitsbelastung in ihrer Abteilung beigetragen haben (Abb. 1c). Es konnten keine Unterschiede zwischen den Berufsgruppen festgestellt werden (Chi-Quadrat-Test; *p* = 0,255). Zwei Teilnehmende (4,0 %) berichteten von einer unzureichenden studentischen Unterstützung aufgrund mangelnder fachlicher Einsatzfähigkeit (*n* = 1) und Routine (*n* = 1) der Hilfskräfte.

### Studentische Überlaufkapazitäten in zukünftigen medizinischen Notlagen

**Abb. 2:**
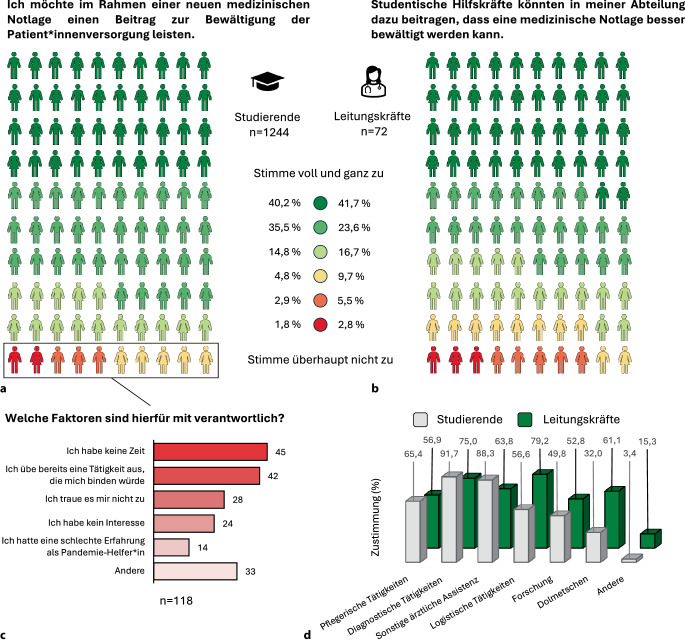
Bereitschaft von Studierenden, an zukünftigen Einsätzen teilzunehmen. **a** Studentische Bereitschaft als Überlaufkapazität in einer potenziellen zukünftigen Notlage, Zustimmung auf einer 6‑Punkte-Likert-Skala, Anteil des jeweiligen Zustimmungswertes in Prozent (k. A. = 5). **b** Anzahl der Studierenden und Gründe für eine fehlende Einsatzbereitschaft, Mehrfachantworten waren möglich. **c** Wahrgenommener Nutzen einer studentischen Überlaufkapazität aus Sicht der Leitungskräfte, Zustimmung auf einer 6‑Punkte-Likert-Skala, Anteil des jeweiligen Zustimmungswertes in Prozent. **d** Zustimmung für die mögliche Delegierbarkeit genannter Tätigkeiten aus Sicht von Studierenden (grau) und Leitungskräften (grün), Zustimmung in Prozent von allen Befragten. Tätigkeitsbeschreibungen im Onlinematerial 1

Im zweiten Abschnitt der Umfrage wurde das zukünftige Interesse zunächst der Studierenden im Hinblick auf einen Einsatz als studentische Helfende evaluiert. 90,5 % der Studierenden (*n* = 1126) stimmten der Aussage: „Ich möchte im Rahmen einer neuen medizinischen Notlage einen Beitrag zur Bewältigung der Patientenversorgung leisten“, zu bzw. voll und ganz zu (M = 5,00, SD = 1,13; Abb. 2a). Es zeigten sich keine Gruppenunterschiede nach Geschlecht (Kruskal-Wallis-Test, *p* = 0,073) und Ausbildungsjahr (*p* = 0,082). 9,5 % der Teilnehmenden (*n* = 118) stimmten der Aussage nicht bzw. überhaupt nicht zu und begründeten dies mit mangelnder Zeit (38,1 %; *n* = 45), Ausübung einer kollidierenden Tätigkeit (35,6 %; *n* = 42) und mangelndem Zutrauen hinsichtlich der auszuübenden Tätigkeit (23,7 %; *n* = 28; Abb. 2b). Bei der Frage nach der Art der Tätigkeit, die sich die Studierenden zutrauen würden, wurden am häufigsten diagnostische Tätigkeiten (91,7 %; *n* = 1032), ärztliche Assistenz (88,3 %; *n* = 994) und pflegerische Tätigkeiten (65,4 %; *n* = 736) genannt (Abb. 2d). Die meisten Tätigkeiten, die in den Freitextantworten genannt wurden, fielen in die Bereiche direkte medizinische Tätigkeiten (68,4 %; *n* = 26; z. B. „Unterstützung in der notfallmedizinischen Versorgung“ oder „ärztliche Tätigkeit, da ich mein Studium bald beendet habe“) oder indirekte medizinische Tätigkeiten (39,5 %, *n* = 15; z. B. „Kommunikationsmittel zwischen Ärzten oder Patienten“; vgl. Onlinematerial 1, Frage 3).

Anschließend wurden die Leitungskräfte zu dem geschätzten Nutzen einer studentischen Überlaufkapazität für zukünftige Notfallsituationen befragt. 81,9 % (*n* = 59) stimmten der Aussage: „Studentische Hilfskräfte könnten in meiner Abteilung/meinem Bereich dazu beitragen, dass eine medizinische Notlage (wie z. B. eine neue Pandemie) besser bewältigt werden kann“, zu bzw. voll und ganz zu (M = 4,77, SD = 1,38; Abb. 2c). Es zeigten sich keine signifikanten Gruppenunterschiede nach Berufsgruppe (*p* = 0,246). Aufgrund der Vielzahl der erhobenen Fachbereiche und der damit verbundenen geringen Fallzahl ist diesbezüglich kein Gruppenvergleich möglich. Auf die Frage nach weiteren, bisher nicht genannten delegierbaren Tätigkeiten wurden logistische Tätigkeiten (79,2 %; *n* = 57), diagnostische Tätigkeiten (75,0 %; *n* = 54) und ärztliche Assistenztätigkeiten (63,9 %; *n* = 46) genannt.

### Rahmenbedingungen für den Einsatz von studentischen Überlaufkapazitäten

Abschließend wurden die Voraussetzungen, Vorteile und Limitationen einer studentischen Überlaufkapazität im Kontext von Preparedness-Strukturen untersucht.

Als notwendige Voraussetzungen für einen zukünftigen Einsatz nannten Studierende eine adäquate finanzielle Entschädigung (77,7 %; *n* = 970), die Klärung in Bezug auf akademische Verpflichtungen, z. B. durch die Anrechnung des Einsatzes als Studienleistung (67,0 %; *n* = 837) oder die Befreiung von Pflichtveranstaltungen (53,6 %; *n* = 670), und eine präemptive Schulung delegierter Tätigkeiten (62,2 %; *n* = 777; Tab. 2). Die Freitextantworten bestätigten die Angaben aus dieser Auswahlfrage.

**Tab. 2:** Rahmenbedingungen sowie potenzielle Vorteile und Limitationen zukünftiger Einsätze aus studentischer Sicht

*Voraussetzungen für die Tätigkeit als Überlaufkapazität*		*Nennungen (Anteil in %)*
Adäquate finanzielle Entschädigung		970 (77,7)
Akademische Anreize, z. B. Anrechnung des Einsatzes als curriculare Leistung		837 (67,0)
Ausbildung in den durchzuführenden Aufgabengebieten im Voraus		777 (62,2)
Befreiung von Pflichtveranstaltungen im Studium		670 (53,6)
Erwerb einer Zertifizierung		422 (33,8)
Regelmäßiges Feedback		400 (32,0)
Begleitung durch Mentorinnen und Mentoren		382 (30,6)
Psychosoziale Begleitung		261 (20,9)
Andere Freitextantworten		42 (3,4)
–	Fachliche Sicherstellung der Einsatzfähigkeit	15 (1,2)
	Keine Nachteile durch den Einsatz	11 (0,9)
	Adäquate räumliche und materielle Ausstattung	6 (0,5)
	Flexible Gestaltung des Einsatzes	4 (0,3)
	Klare arbeitsrechtliche Rahmenbedingungen	3 (0,2)
k. A.		123 (9,8)
*Mögliche Vorteile durch den Einsatz (Freitextantworten)*		*Nennungen (Anteil in %)*
Strukturelle und organisatorische Vorteile (bspw. durch Entlastung der Fachkräfte und Einsatz von fachlichen Vorkenntnissen)		468 (72,1)
Persönliche Vorteile (bspw. durch fachliche Entwicklungsmöglichkeiten, finanzielle oder berufliche Vorteile und durch positive soziale Auswirkungen)		442 (68,1)
*Mögliche Limitationen des Einsatzes (Freitextantworten)*		*Nennungen (Anteil in %)*
Strukturelle und organisatorische Limitationen (bspw. durch akademische Verpflichtungen, ungeklärte Haftungsfragen)		323 (53,3)
Fachliche Limitationen (bspw. durch unzureichende Fachkenntnisse, Schulung o. Supervision)		278 (45,9)
Persönliche Limitationen (bspw. durch private Verpflichtungen, negative finanzielle Auswirkungen o. Gefährdung der eigenen Gesundheit)		218 (36,0)

*k.* *A* keine Angabe/fehlende Werte

Darüber hinaus nannten die Studierenden verschiedene strukturelle und organisatorische Vorteile ihres Einsatzes, z. B. die Entlastung der Fachkräfte, den adäquaten Einsatz ihrer medizinischen Vorkenntnisse (72,1 %; *n* = 468), sowie persönliche Vorteile (68,1 %; *n* = 442), z. B. in Form von persönlichen Entwicklungsmöglichkeiten, finanziellen Anreizen oder beruflichen Vorteilen (Tab. 2).

Einschränkungen ihres Einsatzes sahen die Studierenden in strukturell-organisatorischen Aspekten (53,3 %; *n* = 323), z. B. durch akademische Verpflichtungen und ungeklärte Haftungsfragen, in fachlichen Grenzen (45,9 %; *n* = 278), z. B. durch mangelnde Routine, Kompetenz und Supervision, und in individuellen Faktoren (36,0 %; *n* = 218), z. B. durch familiäre und soziale Verpflichtungen, negative finanzielle oder psychische Auswirkungen (Tab. 2).

Die Studierenden stimmten überwiegend einer präemptiven Vorbereitung auf solche Einsätze während des Studiums zu bzw. voll und ganz zu (63,9 %; *n* = 798; M = 4,76, SD = 1,21). In den Freitextkommentaren gaben sie an, dass sie eine Vorbereitung am liebsten im Rahmen eines freiwilligen curricularen Zusatzangebotes (41,6 %; *n* = 519), z. B. eines longitudinalen Wahlfaches, wünschen. Alternativ nannten die Studierenden eine Vorbereitung als Bestandteil des Kerncurriculums (24,0 %; *n* = 300) oder als extracurriculares Zusatzangebot (23,2 %; *n* = 290).

Die Leitungskräfte nannten für einen reibungslosen Einsatz studentischer Helfender deren suffiziente fachliche Einsatzfähigkeit (54,2 %; *n* = 39). Für die Delegation der in Abb. 2d genannten Tätigkeiten wurden Kenntnisse in Hygienestandards und die Handhabung persönlicher Schutzausrüstung (PSA) gefordert (83,3 %; *n* = 60). Zusätzlich wurden Kenntnisse über typische organisatorische sowie klinische Abläufe (76,4 %; *n* = 55) und eine Einarbeitung in den Einsatzbereich (58,3 %; *n* = 42) aufgezählt (Tab. 3). Darüber hinaus wurden strategisch-operative Rahmenbedingungen erwähnt (27,8 %; *n* = 20), z. B. die adäquate Personalplanung für definierte Einsatzbereiche und dazugehörige detaillierte Tätigkeitsbeschreibungen (z. B.: „Die Aufgaben müssen im Vorfeld dem gesamten Team gegenüber klar formuliert werden. Es dürfen keine Maßnahmen abseits dieses Katalogs durchgeführt werden“, „Strukturierte Aufteilung der Hilfskräfte auf die einzelnen Stationen“). Zuletzt wurde auf die adäquate Personalverwaltung der Hilfskräfte eingegangen (26,4 %; *n* = 14), z. B. die Überwindung administrativer Hürden (z. B.: „Komplette [administrative] Vorbereitung der Hilfskräfte: Berufskleidung, Transponder, Zugang [zum Patientendatenmanagementsystem und zum klinischen Informationssystem]“ und „Finanzierung der Hilfskräfte“; Tab. 3).

**Tab. 3:** Rahmenbedingungen sowie potenzielle Vorteile und Limitationen zukünftiger Einsätze aus Sicht der Leitungskräfte

*Notwendige praktische Kenntnisse für die Einbindung von Studierenden*	*Nennungen (Anteil in %)*
Kenntnisse über den richtigen Einsatz von PSA und Hygienestandards	60 (83,3)
Kenntnisse über typische organisatorische sowie klinische Abläufe	55 (76,4)
Praktische Weiterbildung oder Praktikum in Ihrer Abteilung	42 (58,3)
Kenntnisse über die Dokumentation	40 (55,6)
Vertiefte pflegerische Kenntnisse	27 (37,5)
Andere Freitextantworten (u. a. Geräteeinweisungen, Kommunikationskenntnisse, Laborerfahrung)	9 (12,5)
k. A.	19 (26,4)
*Notwendige Vorbereitung für den reibungslosen Einsatz (Freitextantworten)*	*Nennungen (Anteil in %)*
Fachliche Sicherstellung der Einsatzfähigkeit (bspw. durch Einarbeitungskonzepte, Berücksichtigung von Vorqualifikationen, Bereitstellung von Ressourcen zur Einarbeitung und Supervision)	39 (54,2)
Klare strategische und operative Rahmenbedingungen (bspw. durch Beschaffung von betrieblichen Arbeitsmitteln, EDV-Zugangsberechtigungen und Klärung von akademischen Pflichten der Studierenden)	20 (27,8)
Adäquate Personalverwaltung (bspw. durch die adäquate Personalplanung und Tätigkeitsplanung)	14 (19,4)
k. A.	19 (26,4)
*Vorteile für die Bewältigung einer medizinischen Notlage (Freitextantworten)*	*Nennungen (Anteil in %)*
Entlastung des Stammpersonals (bspw. durch Zuarbeit, Optimierung von Arbeitsabläufen und eigenständige Übernahme diverser Tätigkeiten)	43 (59,7)
Strategische und operative Vorteile (bspw. durch die personelle Verfügbarkeit oder durch das Vorhandensein von Vorkenntnissen)	25 (34,7)
Vorteile für die Studierenden	6 (8,3)
Vorteile für Patienten und Angehörige	4 (5,6)
k. A.	17 (23,6)
*Limitationen eines solchen Einsatzes (Freitextantworten)*	*Anzahl (Anteil in %)*
Strategische und operative Limitationen (bspw. durch Aspekte der Personalplanung, Delegierbarkeit von Aufgaben und die Einbettung in den Strukturen der Klinik)	23 (31,9)
Fachliche Limitationen (bspw. durch mangelhaftes fachliches Wissen oder Einarbeitung)	20 (27,8)
Nachteile für die Studierenden	4 (5,6)
k. A.	24 (33,3)

*PSA* Persönliche Schutzausrüstung; *EDV* elektronische Datenverarbeitung; *k.* *A.* keine Angabe/fehlende Werte

Als Vorteile einer studentischen Überlaufkapazität sehen die Leitungskräfte eine mögliche Entlastung des Stammpersonals (59,7 %; *n* = 43). Darüber hinaus wurden strategisch-operative Vorteile (34,7 %; *n* = 25) genannt, z. B. aufgrund der hohen Verfügbarkeit der Studierenden oder aufgrund fachlicher Vorkenntnisse (z. B.: „geringere Einarbeitungszeit und schnellere Verfügbarkeit durch Ausbildung der studentischen Hilfskräfte“; Tab. 3).

Die Leitungskräfte erwähnten operative Limitationen des Einsatzes (31,9 %; *n* = 23), beispielsweise im Hinblick auf die Personalplanung und die Delegierbarkeit von Aufgaben. Des Weiteren wurden fachliche Limitationen aufgezählt (27,8 %, *n* = 20; z. B. „Geringe praktische Erfahrung. … Wenig Fertigkeiten in der praktischen Arbeit“; Tab. 3).

## Diskussion

In dieser Arbeit wurde der Einsatz von Medizinstudierenden als Hilfskräfte während der COVID-19-Pandemie im Allgemeinen sowohl von den Studierenden als auch von den Leitungskräften als positive Erfahrung bewertet. Beide Gruppen waren gegenüber einem erneuten Einsatz zur Bewältigung einer medizinischen Notlage aufgeschlossen.

Studentische Hilfskräfte werden weltweit zunehmend für medizinische Notlagen eingesetzt und zum Teil systematisch und strukturiert darauf vorbereitet [6, 12]. Unsere multizentrischen Umfragen stellen die bisher größte uns bekannte Erhebung zu einer solchen Überlaufkapazität dar. Im Vergleich zu einer systematischen Übersichtsarbeit unter Studierenden aus 18 Ländern gaben Studierende in dieser Umfrage eine leicht überdurchschnittliche Einsatzbereitschaft an (durchschnittliche Bereitschaft im Review von 68,4 %, Spannweite: 26,7–87,8 %; [13]).

Unsere Studierendenumfrage hatte das Potenzial, bis zu etwa 45.500 Studierende zu erreichen, und hatte einen Rücklauf von 3 %. Dies ist wahrscheinlich darauf zurückzuführen, dass die kontaktierten Fakultäten unterschiedliche Verbreitungsmethoden (z. B. digitale Schwarze Bretter oder E‑Mail) gewählt haben, welche eine unterschiedliche Reichweite aufweisen können. Zum Vergleich erzielte eine bundesweite Sozialerhebung bei 1.397.326 Studierenden von 250 Hochschulen 2021 eine Teilnahmequote von 13 % [14]. Dennoch konnte eine hohe absolute Zahl an Rückmeldungen zur untersuchten Fragestellung eingeholt werden. Wie bereits in ähnlichen Umfragen berichtet, kann nicht ausgeschlossen werden, dass ein Selbstselektionsbias aufgrund polarisierender Meinungen zu diesem Thema die erhobenen Daten verzerrt [13]. Aufgrund der strengen Datenschutzbestimmungen konnte die Leitungskräfteumfrage an weniger Standorten als geplant durchgeführt werden, was zu einer geringeren absoluten Teilnehmerzahl führte. Mit 33 % war die Rücklaufquote für eine Online-Befragung zufriedenstellend. Die Teilnehmenden repräsentierten ein breites Spektrum unterschiedlicher medizinischer Disziplinen. Dennoch wiesen die Ergebnisse eine hohe inhaltliche Kongruenz auf.

Ein wesentlicher Aspekt dieser Arbeit stellte die Benennung von Rahmenbedingungen dar, welche einen Preparedness-Einsatz möglich machen würden. Diese Erhebungen zeigen, dass Überlaufkapazitäten sorgfältig geplant werden sollten, um limitierende Faktoren möglichst im Vorfeld zu adressieren. Als essenzieller Faktor wurde die fachliche Sicherstellung der Einsatzfähigkeit benannt. Eine systematische Übersichtsarbeit zum Einsatz von Studierenden in Notlagen zeigte, dass Medizinstudierende in vielen Ländern über unzureichendes Wissen in Bezug auf die Bewältigung von Notlagen verfügen [13]. In unserer Umfrage forderten beide Gruppen eine präemptive fachliche Einarbeitung der Studierenden für mögliche zukünftige Einsätze. Hierzu wurden bereits im Rahmen der COVID-19-Pandemie Schulungskonzepte vorgeschlagen, die für zukünftige Notlagen berücksichtigt werden könnten [15, 16]. Die starke Überschneidung zwischen der Befragung beider Parteien hinsichtlich möglicher delegierbarer Tätigkeiten zeigt, dass eine präemptive Tätigkeitsbeschreibung möglich sein sollte. Die in der Studierendenumfrage bevorzugte Vorbereitung in Form eines freiwilligen Zusatzangebotes für Medizinstudierende (z. B. als longitudinales Wahlfach) würde die Rekrutierung und präemptive Schulung besonders motivierter Studierenden ermöglichen. Die Ausweitung einer studentischen Überlaufkapazität auf andere Studiengängen (z. B. Pharmazie oder Gesundheitswissenschaften) erscheint in Betracht vorbeschriebener Konzepte möglich, jedoch für den klinischen Einsatz in der Patientenversorgung unter Umständen mit vermehrtem Schulungsaufwand assoziiert [6]. Der Einsatz von Studierenden und Auszubildenden der Pflege- und Therapieberufe wurde bereits während der COVID-19-Pandemie vorgeschlagen und Auflagen zur flexibleren Gestaltung ihrer Ausbildung festgelegt, um Ausbildung und praktische Einsätze in Krisenzeiten zu ermöglichen [17, 18]. Aktuelle Bemühungen im Katastrophenschutz beinhalten neue Schulungskonzepte mit Inhalten aus der Krisen- und Katastrophenbewältigung für Pflegefachkräfte und Auszubildende. Diese sollen im Verlauf in die generalistische Pflegeausbildung eingebettet werden [19].

Diese Arbeit erhob die Perspektiven von Medizinstudierenden sowie klinisch tätigen Leitungskräften. Die Perspektiven anderer Akteure, welche in der Erstellung von Preparedness-Plänen und in der Bewältigung von medizinischen Notlagen involviert sind, wurden in dieser Arbeit nicht berücksichtigt. Die erwünschten Rahmenbedingungen für diese Überlaufkapazität haben Konsequenzen für den Gesetzgeber, beispielsweise bei der Bereitstellung von finanziellen Ressourcen für die Schulung und Entschädigung von Hilfskräften. Zusätzlich muss die Perspektive von Patientenvertretern in Bezug auf Aspekte der Patientensicherheit beachtet werden. Die Planung von Hilfskräften für Zeiten des absoluten oder relativen Fachkräftemangels erfolgt hauptsächlich im Sinne der Patientensicherheit. Hier ist anzunehmen, dass aus Sicht der Patientenvertreter eine möglichst ausführliche fachliche Vorbereitung dieser Hilfskräfte erwünscht wäre. Zusätzlich müssen zeitnah unter anderem hochschulpolitische und approbationsrechtliche Aspekte eines solchen Einsatzes geklärt werden, z. B. im Umgang mit akademischen Verpflichtungen. Der versicherungsrechtliche Aspekt eines solchen Einsatzes ist im Kontext anderer studentischer Einsätze im Studium als ausreichend geklärt beschrieben worden [15].

## Fazit

Zusammenfassend unterstützen diese Ergebnisse den geplanten und strukturierten Einsatz von Medizinstudierenden als Überlaufkapazitäten an deutschen Universitätskliniken und bilden die Grundlage für die Entwicklung eines einheitlichen Konzeptes für diese Personalressource im Rahmen der von der PREPARED Working Group erarbeiteten Preparedness-Pläne.

## Supplementary Information

ESM 1 Fragebögen

ESM 2 Codierleitfaden

ESM 3 Studentische Teilnehmer nach Fakultät

## Data Availability

Die in dieser Studie erhobenen Datensätze sind auf begründete Anfrage bei der Korrespondenzperson erhältlich.
