# Supplementary material for: Medical students in hospital emergency preparedness in the wake of the COVID-19 pandemic: a qualitative analysis of the ESCAPE study
Source: Bundesgesundheitsblatt Gesundheitsforschung Gesundheitsschutz. 2026 May 4;69(6):666–75. [Article in German] doi: 10.1007/s00103-026-04236-4 (PMC13212819; doi:10.1007/s00103-026-04236-4)
Supplement: Supplementary file 1 — ESM 1 Fragebögen [file 103_2026_4236_MOESM1_ESM.pdf]

Onlinematerial 1: Umfragen

Studierendenumfrage

1. Willkommen zur Umfrage "pandemic preparedness" aus Studierendenperspektive

Liebe Studierende,

vielen Dank für Ihr Interesse an unserer Studie "ESCAPE - Evaluating Surge Capacity And PrEparedness".

Im Rahmen dieser Studie soll untersucht werden, ob für die Bewältigung einer zukünftigen medizinischen Notlage (wie z.B. eine neue Epidemie/Pandemie) der Einsatz ausgebildeter studentischer Hilfskräfte als Überlaufkapazität (surge capacity) eine Entlastung für die Patient*innenversorgung an (Universitäts-) Kliniken darstellen kann.

Die Studie wird durch das Bundesministerium für Bildung und Forschung (BMBF)[1] im Rahmen von PREPARED des Netzwerks Universitätsmedizin (NUM 2.0) gefördert. Durchgeführt wird sie von der Klinik für Anaesthesiologie und dem Institut für Didaktik und Ausbildungsforschung in der Medizin am LMU Klinikum in Kooperation mit der Universitätsmedizin Hannover, Leipzig und Lübeck.

Die Umfrage ist anonym und freiwillig. Sie können die Umfrage jederzeit abbrechen. Es entstehen keinerlei Vor- oder Nachteile bei Teilnahme oder Nicht-Teilnahme. Die Studie wurde durch die Ethikkommission der LMU München sowie durch den Datenschutzbeauftragten genehmigt.

2. Demographische Informationen und COVID-19

2.1. Welchem Geschlecht fühlen Sie sich zugehörig?

❑ weiblich ❑ männlich ❑ divers ❑ k.a.

2.2. In welchem Jahr sind Sie geboren? (Z. B. 1998)
*(Freitextantwort)*________________________________

2.3. An welcher medizinischen Fakultät sind Sie immatrikuliert?
(Mehrfachantwort möglich)

❑ RWTH Aachen University
❑ Universität Augsburg
❑ Charité - Universitätsmedizin Berlin
❑ Ruhr-Universität Bochum
❑ Universität Bonn
❑ Universität Bonn-Siegen
❑ Technische Universität Dresden
❑ Universität Duisburg-Essen
❑ Universität Düsseldorf
❑ Friedrich-Alexander- Universität Erlangen-Nürnberg

[1] Zum Zeitpunkt der Publikation als Bundesministerium für Forschung, Technologie und Raumfahrt (BMFTR) bezeichnet

❑ Goethe-Universität Frankfurt

❑ Albert-Ludwigs-Universität Freiburg

❑ Justus-Liebig-Universität Gießen

❑ Georg-August-Universität Göttingen

❑ Martin-Luther-Universität Greifswald

❑ Martin-Luther-Universität Halle-Wittenberg

❑ Universität Hamburg

❑ Medizinische Hochschule Hannover

❑ Ruprecht-Karls-Universität Heidelberg - Medizinische Fakultät Heidelberg

❑ Ruprecht-Karls-Universität Heidelberg - Medizinische Fakultät Mannheim

❑ Friedrich-Schiller-Universität Jena

❑ Christian-Albrechts-Universität zu Kiel

❑ Universität zu Köln

❑ Universität Leipzig

❑ Universität zu Lübeck

❑ Otto-von-Guericke-Universität Magdeburg

❑ Johannes Gutenberg- Universität Mainz

❑ Philipps-Universität Marburg

❑ Ludwig-Maximilians- Universität München

❑ Technische Universität München

❑ Westfälische Wilhelms- Universität Münster

❑ Carl von Ossietzky Universität Oldenburg

❑ Universität Regensburg

❑ Universität Rostock

❑ Universität des Saarlandes

❑ Eberhard Karls Universität Tübingen

❑ Universität Ulm

❑ Julius-Maximilians-Universität Würzburg

2.4. In welchem Fachsemester befinden Sie sich aktuell?

❑ 1	❑ 2	❑ 3	❑ 4
❑ 5	❑ 6	❑ 7	❑ 8
❑ 9	❑ 10	❑ 11	❑ 12
❑ 13	❑ 14	❑ 15	❑ 16

2.5. Haben Sie im Laufe der COVID-19 Pandemie als "Pandemie-Helfer*in" in der Versorgung von Patient*innen mitgewirkt?

❑ Ja, ich war im klinischen Bereich tätigt.

❑ Ja, ich war in einem nicht-klinischen Bereich tätig (bspw. Gesundheitsamt, Test-

❑ Nein

Zentrum, Impf-Zentrum, usw.).

2.6. In welchem Bereich der Patient*innenversorgung waren Sie tätig? *(Freitextantwort)*____

2.7. Inwieweit stimmen Sie der folgenden Aussage zu:
Ich hatte das Gefühl, dass ich mit meinem Einsatz als Pandemie-Helfer*in einen sinnvollen Beitrag zur Bewältigung der Personalknappheit während der Pandemie leisten konnte.

Stimme überhaupt nicht zu ❑ ❑ ❑ ❑ ❑ ❑ Stimme voll und ganz zu

2.8. Warum haben Sie das Gefühl, dass Ihre Arbeit als nicht hilfreich wahrgenommen wurde?
*(Freitextantwort)*____

3. Zukünftige medizinische Notlagen

3.1. Inwieweit stimmen Sie der folgenden Aussage zu:
Ich möchte im Rahmen einer neuen medizinischen Notlage einen Beitrag zur Bewältigung der Patient*innenversorgung leisten.

Stimme überhaupt nicht zu ❑ ❑ ❑ ❑ ❑ ❑ Stimme voll und ganz zu

3.2. Wenn Sie nicht bereit sind, an einem solchen Einsatz teilzunehmen, welche Faktoren sind hierfür mit verantwortlich? *(Mehrfachantwort möglich)*

❑ Ich habe keine Zeit

❑ Ich habe kein Interesse

❑ Ich habe im Rahmen der COVID-19 Pandemie eine schlechte Erfahrung als Pandemie-Helfer*in gemacht

❑ Ich übe bereits eine Tätigkeit aus, die mich im Rahmen einer medizinischen Notlage binden würde

❑ Ich traue es mir nicht zu

❑ Andere: ______________________

3.3. Welche Form des Einsatzes würden Sie sich zutrauen? *(Mehrfachantworten möglich)*

❑ Pflegerische Tätigkeiten

❑ Sonstige ärztliche Assistenz (z.B. Vorbereitung von diagnostischen oder therapeutischen Maßnahme)

❑ Unterstützung bei der Durchführung pandemiebedingter Forschungsfragen

❑ Diagnostische Tätigkeiten (Blutentnahmen, Point-of-care Analysen, Abstriche, Bearbeitung von Proben in der Laboratoriumsmedizin oder Mikrobiologie, u.v.m.)

(Organisation, Logistik, Datensammlung und - auswertung)

❑ Logistische Tätigkeiten (Patient*innentransport, Kontakt-Nachverfolgung usw.)

❑ Dolmetschen

❑ Keine

❑ Andere: ______________________

3.4. Welche Voraussetzungen müssten erfüllt sein, damit Sie diese Aufgaben - selbstverständlich nach Klärung aller haftungs- und versicherungsrechtlichen Fragen - übernehmen würden? *(Mehrfachantworten möglich)*

❑ Ausbildung in den durchzuführenden Aufgabengebieten im Voraus

❑ Adäquate Entlohnung der Tätigkeit

❑ Befreiung von Pflichtveranstaltungen im Studium und ggf. Verschieben von Klausuren

❑ Anreize, z.B. durch die Anrechnung des Einsatzes im Rahmen des Studiums (als Famulatur o.Ä.)

❑ Belohnung durch Erwerb einer Zertifizierung

❑ Begleitung durch Mentor*innen

❑ Psychosoziale Begleitung

❑ Regelmäßiges Feedback

❑ Andere: ______________________

3.5. Welche Vorteile sehen Sie, wenn Sie als ausgebildete studentische Hilfskraft zur Bewältigung einer potenziellen medizinischen Notlage eingesetzt würden? *(Freitext)*____

3.6. Welche Limitation sehen Sie in einem solchen Einsatz? *(Freitext)*____

3.7. In welcher Form würden Sie auf einen solchen Einsatz vorbereitet werden wollen?

❑ Als fester Bestandteil des Curriculums

❑ Als freiwilliges curriculares Zusatzangebot (z.B. longitudinales Wahlfach über mehrere Semester)

❑ Als extracurriculäres Zusatzangebot

3.8. Inwieweit stimmen Sie der folgenden Aussage zu:
Es ist grundsätzlich sinnvoll, Studierende in Vorbereitung auf eine zukünftige medizinische Notlage (z.B. eine Pandemie) begleitend zum Studium spezifisch zu schulen.

Stimme überhaupt nicht zu ❑ ❑ ❑ ❑ ❑ ❑ Stimme voll und ganz zu

3.9. Wären Sie bereit, dem Dekanat zur zielgerichteteren Rekrutierung im Bedarfsfall eventuell bereits vorliegende persönliche Qualifikationen (wie z. B.

Vorliegen einer Berufsausbildung als Gesundheits- und Krankenpfleger*in oder präklinische Erfahrung) mitzuteilen?

❑ Ja ❑ Nein ❑ Weiß ich nicht

Leitungskräfteumfrage

1. Willkommen zur Befragung von Mitarbeitenden im Hinblick auf "pandemic preparedness"

   Liebe Kolleg*innen,
   vielen Dank für Ihr Interesse an unserer Studie "ESCAPE - Evaluating Surge Capacity And PrEparedness".

   Im Rahmen dieser Studie soll untersucht werden, ob für die Bewältigung einer zukünftigen medizinischen Notlage (wie z. B. eine neue Epidemie/Pandemie) der Einsatz ausgebildeter studentischer Hilfskräfte als Überlaufkapazität (surge capacity) eine Entlastung für die Patient*innenversorgung an (Universitäts-) Kliniken darstellen kann.

   Zusätzlich sollen Aufgaben ausgearbeitet werden, die von Studierenden bei einer zukünftigen medizinischen Notlage übernommen werden könnten und Kompetenzen definiert werden, die geschult werden sollten.

   Die Studie wird durch das Bundesministerium für Bildung und Forschung (BMBF)[1] im Rahmen von PREPARED des Netzwerks Universitätsmedizin (NUM 2.0) gefördert. Durchgeführt wird sie von der Klinik für Anaesthesiologie und dem Institut für Didaktik und Ausbildungsforschung in der Medizin am LMU Klinikum in Kooperation mit der Universitätsmedizin Hannover, Leipzig und Lübeck.

   Die Umfrage ist anonym und freiwillig. Sie können die Umfrage jederzeit abbrechen. Es entstehen keinerlei Vor- oder Nachteile bei Teilnahme oder Nicht-Teilnahme. Die Studie wurde durch die Ethikkommission der LMU München, den Personalrat sowie durch den Datenschutzbeauftragten genehmigt.

2. Demographische Angaben

   2.1 In welchem Bereich sind Sie tätig?

❑ Nothilfe	❑ Intensivstation / Intermediate Care Unit	❑ Allgemeinstation – internistisch
❑ Allgemeinstation – chirurgisch	❑ Allgemeinstation – anderes Fachgebiet	❑ Funktionsdienst
❑ Pädiatrie	❑ Mikrobiologie	❑ Stabsstelle antibiotic stewardship (ABS-Team)
❑ Laboratoriums-medizin	❑ Hygiene	❑ Patienten-transportdienst
❑ Forschung	❑ Andere: ____________________	

   2.2 Ordnen Sie bitte Ihre berufliche Rolle ein *(Mehrfachantwort möglich)*:

❑ Pflege	❑ Ärzteschaft	❑ Technische Assistenz
❑ Stationsleitung	❑ Oberärztliche Funktion	❑ Sonstige Leitungsfunktion

❑ Andere: ______________________________________

3. Einsatz ausgebildeter studentischer Hilfskräfte

3.1. Wurden während der COVID-19-Pandemie studentische Hilfskräften in Ihrer Abteilung / Ihrem Bereich eingesetzt?

❑ Ja ❑ Nein ❑ Weiß ich nicht

3.2. War dieser Einsatz eine Entlastung für die Bewältigung Ihrer Arbeit?

❑ Ja ❑ Nein ❑ keine Angabe

3.3. Warum war dieser Einsatz nicht hilfreich?
*(Freitext)*____

3.4. Inwieweit stimmen Sie folgender Aussage zu:
Studentische Hilfskräfte könnten in meiner Abteilung / meinem Bereich dazu beitragen, dass eine medizinische Notlage (wie z. B. eine neue Pandemie) besser bewältigt werden kann.

Stimme überhaupt nicht zu ❑ ❑ ❑ ❑ ❑ ❑ Stimme voll und ganz zu

3.5. Die Übertragung folgender Aufgaben oder Prozesse auf ausgebildete studentische Hilfskräfte könnte in einer medizinischen Notlage die Mitarbeitenden Ihrer Abteilung entlasten *(Mehrfachantworten möglich)*:

❑ Pflegerische Tätigkeiten

❑ Sonstige ärztliche Assistenz (z.B. Vorbereitung von diagnostischen oder therapeutischen Maßnahme)

❑ Unterstützung bei der Durchführung pandemiebedingter Forschungsfragen (Organisation, Logistik, Datensammlung und - auswertung)

❑ Dolmetschen

❑ Diagnostische Tätigkeiten (Blutentnahmen, Point-of-care Analysen, Abstriche, Bearbeitung von Proben in der Laboratoriumsmedizin oder Mikrobiologie, u.v.m.)

❑ Logistische Tätigkeiten (Patient*innentransport, Kontakt-Nachverfolgung usw.)

❑ Andere: ______________________

3.6. Folgende praktische Kenntnisse wären Voraussetzungen für die Übertragung dieser Aufgaben auf studentische Hilfskräfte in Ihrer Abteilung / Ihrem Bereich *(Mehrfachantworten möglich)*:

❑ Vertiefte pflegerische Kenntnisse

❑ Kenntnisse über die Dokumentation

❑ Praktische Weiterbildung oder Praktikum in Ihrer Abteilung

- [ ] Kenntnisse über den richtigen Einsatz von persönlicher Schutzausrüstung (PSA) und Hygienestandards
- [ ] Kenntnisse über typische organisatorische sowie klinische Abläufe
- [ ] Andere: ______________________

3.7. Welche Vorbereitung und Unterstützung bräuchte Ihre Abteilung / Ihr Bereich, damit der Einsatz zusätzlicher studentischer Hilfskräfte möglichst reibungslos ablaufen kann?
*(Freitext)*____

3.8. Welche Vorteile sehen Sie im Einsatz ausgebildeter studentischer Hilfskräfte in der Bewältigung einer medizinischen Notlage in Ihrer Abteilung?
*(Freitext)*____

3.9. Welche Limitation sehen Sie für einen solchen Einsatz?
*(Freitext)*____

3.10. Gibt es noch etwas, dass Sie uns zum Thema "Einsatz studentischer Hilfskräfte als Überlaufskapazität im Rahmen einer zukünftigen medizinischen Notlage" mitteilen möchten:
*(Freitext)*____
