# Supplementary material for: Medical students in hospital emergency preparedness in the wake of the COVID-19 pandemic: a qualitative analysis of the ESCAPE study
Source: Bundesgesundheitsblatt Gesundheitsforschung Gesundheitsschutz. 2026 May 4;69(6):666–75. [Article in German] doi: 10.1007/s00103-026-04236-4 (PMC13212819; doi:10.1007/s00103-026-04236-4)
Supplement: Supplementary file 2 — ESM 2 Codierleitfaden [file 103_2026_4236_MOESM2_ESM.pdf]

Codierleitfaden Studierendenumfrage

In welchem Bereich der Patient*innenversorgung waren Sie tätig?

Kategorie	Definition	Ankerbeispiel	Codierregel
K1: Bereiche der klinischen Patientenversorgung	Klinische Bereiche, die sich teilweise oder ganz mit der stationären Patientenversorgung beschäftigen. Bspw: Notaufnahme, Intensivstation, IMC, Normalstation	Notaufnahme, Intensivstation, IMC, Normalstation, Operationsbereich	Es wird mindestens ein Tätigkeitsbereich in der stationären Patientenversorgung beschrieben.
K2: Bereiche der Infektionsprävention	Medizinische Tätigkeitsbereiche, die sich mit infektiologischer Prävention und Diagnostik beschäftigen.	Impfzentrum, Teststation, Test-Labor, Infektiologie	Es wird mindestens ein Tätigkeitsbereich in Strukturen der Infektionsprävention (primäre oder sekundäre Prävention) beschrieben.
K3: Bereiche der ambulanten Patientenversorgung	Medizinische Tätigkeitsbereiche in der ambulanten Patientenversorgung	Ambulante medizinische Versorgung, Krankentransportdienst, Rettungsdienst	Es wird mindestens ein Tätigkeitsbereich in der ambulanten Patientenversorgung beschrieben.
K4: Klinische Funktionsbereiche	Medizinische Funktionsbereiche, die sich nicht mit der stationären oder ambulanten Patientenversorgung beschäftigen, und die keinen Beitrag zur infektiologischen Prävention oder Diagnostik leisten.	Radiologie, Laboratoriumsmedizin	Es wird mindestens eine Tätigkeit in einem klinischen Funktionsbereich beschrieben. Ausgenommen sind Bereiche, die unter Kategorie K2 einzuordnen sind.
K5: nicht-klinische Tätigkeitsbereiche		Innerklinischer Patiententransport, Gesundheitsamt, Sicherheitsdienst	Es wird mindestens eine nicht-klinische Tätigkeit beschrieben, die nicht unter den o.g. Kategorien fällt.

Warum haben Sie das Gefühl, dass Ihre Arbeit als nicht hilfreich wahrgenommen wurde?

Kategorie	Definition	Ankerbeispiel	Codierregel
K1: Keine fachliche Sicherstellung der Einsatzfähigkeit	Die Studierenden empfanden, dass sie für den Einsatz fachlich unzureichend einsatzfähig waren, bspw. durch mangelnder Schulung oder Supervision.	"da ich keine ausgebildete Pflegekraft bin, konnte ich viele Aufgaben nicht alleine (ohne Aufsicht) durchführen."	Die Antwort weist auf eine mangelnde fachliche Sicherstellung der Einsatzfähigkeit der Studierenden hin.
K2: Falscher Einsatz personeller Ressourcen	Die Studierenden empfanden, dass die personelle Ressource, die sie darstellen, inadäquat eingesetzt wurde, bspw. durch Fehlverteilung, mangelnde Arbeitsbelastung oder durch nicht-Beachten individueller Fähigkeiten.	"Als Rett. Ass. Mit fast 10 Jahren Berufserfahrung auch im Intensivtransport, mit einschlägigen Fortbildungen, Gepäck auf die Stationen bringen, während 2. Semester Studenten frisch nach dem Abitur auf Intensivstation eingesetzt werden ist eine Verschwendung von Ressourcen"	Die Antwort weist auf einen inadäquaten Einsatz der Studierenden als personelle Ressource hin.
K0: Antwort nicht zuordenbar	Die Antwort ist nicht verständlich formuliert oder beantwortet die o.g. Frage nicht.		Die Antwort ist unklar formuliert oder geht auf eine andere Fragestellung ein.

Wenn Sie nicht bereit sind, an einem solchen Einsatz teilzunehmen, welche Faktoren sind hierfür mit verantwortlich? Andere:

Kategorie	Definition	Ankerbeispiel	Codierregel
K1: Persönliche Faktoren	Faktoren, die direkt von den Studierenden ausgehen oder sich direkt durch den Einsatz auf sie persönlich auswirken, führen zu einer fehlenden Einsatzbereitschaft. Hierzu zählen bspw. zeitliche, gesundheitliche oder finanzielle Einschränkungen.	"Die Bezahlung ist für das Infektionsrisiko zu schlecht"	Die Antwort weist auf persönliche Gründe hin, die einem Einsatz im Wege stehen würden, und nicht unter Kategorie K2 einzuordnen sind.
K2: Strukturelle und organisatorische Faktoren	Strukturelle Faktoren, die die Einbettung der Studierende im Tätigkeitsumfeld beeinflussen, führen zu einer fehlenden Einsatzbereitschaft. Hierzu gehören u.a. arbeitsrechtliche Faktoren, sozialrechtliche Faktoren sowie akademische Faktoren.	"Im Rahmen der Corona-Pandemie hat das durch Ansteckung zu Fehlzeiten geführt, die von der Uni nicht kulant gehandhabt wurden."	Die Antwort weist auf nicht-persönliche Faktoren hin, die die Einsatzbereitschaft hindern.
K3: Fachliche Faktoren	Faktoren, die die fachliche Einsatzfähigkeit der Studierenden beeinflussen, führen zu eine fehlende Einsatzbereitschaft. Hierzu zählen unzureichendes Fachwissen, Fachkompetenz oder Eigenständigkeit in der Versorgung von Patienten.	"Zum aktuellen Zeitpunkt glaube ich nicht, dass Studierende der Aufgabe innerklinisch dazu in der Lage sind auf Grund ihres Ausbildungsstandes entsprechend gut zu unterstützen."	Die Antwort weist auf Faktoren hin, die eine Auswirkung auf die fachliche Einsatzfähigkeit der Studierenden haben.
K0: Antwort nicht zuordenbar	Die Antwort ist nicht verständlich formuliert oder beantwortet die o.g. Frage nicht.		Die Antwort ist unklar formuliert oder geht auf eine andere Fragestellung ein.

Welche Form des Einsatzes würden Sie sich zutrauen ? Andere:

Kategorie	Definition	Ankerbeispiel	Codierregel
K1: Direkte medizinische Tätigkeiten	Medizinische Tätigkeiten, die einen physisch Patientenkontakt bedürfen.	"Impfungen"; "Physiotherapeutische Behandlung"	Die Antwort weist auf die mögliche Übernahme von medizinischen Tätigkeiten direkt am Patienten hin.
K2: Indirekte medizinische Tätigkeiten	Medizinische Tätigkeiten die keinen physischen Patientenkontakt bedürfen, bspw. Aufklärungstätigkeiten.	"Aufklärung der Bevölkerung"	Die Antwort weist auf die mögliche Übernahme von medizinischen Tätigkeiten, die ohne physischen Patientenkontakt erledigt werden können.
K3: nicht-medizinische Tätigkeiten	Tätigkeiten, die nicht zur Patientenversorgung im medizinischen Sinne beitragen.	"Organisationsaufgaben, z.B. Organisation eines Teststellenaufbaus, etc."; "z.B. mit IT Infrastruktur"	Die Antwort weist auf die mögliche Übernahme von Tätigkeiten hin, die keine direkte Auswirkung auf die Patientenbehandlung aufweisen.

Welche Voraussetzungen müssten erfüllt sein, damit Sie diese Aufgaben übernehmen würden? Andere:

Kategorie	Definition	Ankerbeispiel	Codierregel
K1: Fachliche Sicherstellung der Einsatzfähigkeit	Die Einsatzfähigkeit sollte fachlich sichergestellt werden, bspw. durch eine adäquate Schulung und / oder Supervision durch erfahrenen Mitarbeitenden.	"adäquate Anleitung und Einarbeitung, bzw. Möglichkeit für Rückfragen"; "Anlernen der Tätigkeit im Voraus";	Die Antwort weist auf die Notwendigkeit einer fachlicher Sicherstellung der Einsatzfähigkeit hin.
K2: Adäquate räumliche, materielle und Schutzausstattung	Für den Einsatz sollten eine adäquate räumliche Ausstattung sichergestellt sein. Arbeitsmittel sollte zur Verfügung stehen, bspw. persönliche Schutzausrüstung und Schutzimpfungen.	"Arbeitsplatz (Büro-Raum)"; "Ausreichende Maßnahmen zum Eigenschutz […]"	Die Antwort weist auf die Notwendigkeit von Arbeitsmitteln, räumlicher Ausstattung und Schutzaustattung hin.
K3: Klare arbeitsrechtliche Rahmenbedingungen	Der Einsatz sollte arbeitsrechtlich klar definiert sein, bspw. durch Klärung vertragrechtlicher oder haftungsrechtlicher Fragen.	"Schriftlicher Vertrag und Entlohnung"	Die Antwort weist auf die Notwendigkeit klarer arbeitsrechtlicher Rahmenbedingungen im Einsatz hin.
K4: Flexible Gestaltung des Einsatzes	Der Einsatz sollte durch die Studierende flexibel gestaltet werden. Hierzu zählen bspw. die Auswahl des Tätigkeitsgebietes, die durchgeführte Tätigkeiten oder die zeitliche Gestaltung.	"Flexible Arbeitsmöglichkeiten (keine mindeststundenzahl)"	Die Antwort weist auf den Wunsch nach Gestaltungsmöglichkeit im Einsatz durch die Studierenden hin.
K5: Keine Nachteile durch den Einsatz	Der Einsatz sollte kein Nachteil im privaten oder öffentlichen Leben mit sich bringen, bspw. durch akademische Nachteile oder finanzielle Nachteile.	"Sicherstellung, dass die Partizipation keine Nachteile im Studium nach sich zieht […]"; "Verlängerung BaföG-Bewilligung […]"	Die Antwort weist auf Notwendigkeit hin, für den Einsatz limitierende Faktore auszuschalten.
K0: Antwort nicht zuordenbar	Die Antwort ist nicht verständlich formuliert oder beantwortet die o.g. Frage nicht.		Die Antwort ist unklar formuliert oder geht auf eine andere Fragestellung ein.

Welche Vorteile sehen Sie, wenn Sie als ausgebildete studentische Hilfskraft zur Bewältigung einer potenziellen medizinischen Notlage eingesetzt würden?

Kategorie	Definition	Ankerbeispiel	Codierregel
K1: Persönliche Vorteile	Der Einsatz würde positive Auswirkungen auf den Studierenden haben, bspw. durch Entwicklungsmöglichkeiten oder durch finanzielle Vorteile.	"Fachliche aber auch vor allem soziale Erfahrungen, welche einen wertvollen Beitrag zur Persönlichkeitsentwicklung beitragen"; "Man kann Studiennah zusätzliches Geld verdienen und Erfahrung sammeln"; "Für mich natürlich bereits klinische Erfahrung und Patientenkontakt. Knüpfen von Kontakten, Kennenlernen von Kliniken etc"	Die Antwort weist auf einen Mehrwert eines Einsatzes für die Studierenden hin.
K2: Strukturelle und organisatorische Vorteile	Der Einsatz von Medizinstudierenden an der Stelle anderer Personalquellen würde sich nach extern positiv auswirken, bspw. auf die klinische Arbeitslast.	"Großes Kontingent von flexibel einsetzbaren Kräften, die sich durch Studium, Famulatur oder Nebenjob oft bereits örtlich und in den IT-Systemen auskennen ohne dass diese Kräfte an anderer Stelle abgezogen würden."; "Für die Arbeit: möglicherweise relevante Vorkenntnisse werden schon mitgebracht."; Kein oder wenig Einarbeitung notwendig Man ist mit gewissen Aufgaben besser vertraut"	Die Antwort weist auf Vorteile, die sich auf anderen Ebenen außer der persönlichen Ebene der Studierenden auswirkt.
K0: Antwort nicht zuordenbar	Die Antwort ist nicht verständlich formuliert oder beantwortet die o.g. Frage nicht.		Die Antwort ist unklar formuliert oder geht auf eine andere Fragestellung ein.

Welche Limitation sehen Sie in einem solchen Einsatz?

Kategorie	Definition	Ankerbeispiel	Codierregel
K1: Persönliche Limitationen	Der Einsatz wäre limitiert durch Faktoren, die direkt von den Studierenden ausgehen oder sich direkt auf sie auswirken. Hierzu zählen bspw. Einschränkungen durch das Privatleben oder Auswirkungen auf das Privatleben, auf die Gesundheit oder auf die eigenen Finanzen. Ausgenommen sind Faktoren die durch akademische Verpflichtungen zustande kommen.	"psychische und körperliche Belastung"; "Ansteckungsgefahr, Ansteckung der eigenen Familie/ Mitbewohner:innen"	Die Antwort weist auf eine oder mehr Limitationen, die von Studierenden selbst ausgehen oder sich auf sie persönlich auswirken und nicht in Kategorie K2 einzuordnen sind.
K2: Strukturelle und organisatorische Limitationen	Der Einsatz wäre durch organisationelle Gegebenheiten limitiert, die dazu führen, dass der Ablauf des Einsatzes oder die Durchführung der Tätigkeit gehindert wären. Organisationelle-strukturelle Gegebenheiten beinhalten u.a. die universitäre Verpflichtungen der Studierenden, die bürokratischen Abläufe, die zur Einstellung und Verwaltung der Studierende beitragen, die Verwaltung der Studierende am Arbeitsplatz und die Klärung von Haftungsfragen.	"begrenzte Zeit während des Semesters um das eigene Studium nicht zu vernachlässigen"; "ggf. Nachteile im Studium durch die aufgebrachte Zeit"; "Eingeschränktes Wissen, eingeschränkte Fähigkeiten, fehlende rechtliche Absicherung, keien Zeit parallel zum Studium."	Die Antwort weist auf strukturelle Hürden, die den Einsatz der Studierende limitieren würden.

Welche Limitation sehen Sie in einem solchen Einsatz?

K3: Fachliche Limitationen	Der Einsatz wäre durch das fachliche Wissen der Studierenden, oder Mangel davon limitiert. Hierzu gehören bspw. unzureichendes Vorwissen, unzureichende Schulung und Supervision.	"Als Student wird man während die Studium oft nicht gut betreut bzw. werden ärztliche Tätigkeiten einen nicht gut vorbeigebracht. Meiner Meinung nach ist für eine effektiven Einsatz von studentischen Hilfskräfte bei einer Notfallsituation die ausführlichen Schulung von den Studenten essentiell. Sonst würde man mehr Schaden als gut tun."	Die Antwort weist auf fachliche Mängel hin, die die Übernahme von Tätigkeiten verhindern würde oder zu ein unzuverlässiges Erfühlen der Tätigkeit führen würde.
K0: Antwort nicht zuordenbar	Die Antwort ist nicht verständlich formuliert oder beantwortet die o.g. Frage nicht.		Die Antwort ist unklar formuliert oder geht auf eine andere Fragestellung ein.

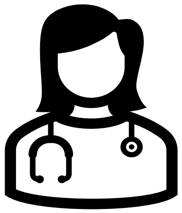

Codierleitfaden Leitungskräfteumfrage

In welchem Bereich der Patient*innenversorgung waren Sie tätig?

Kategorie	Definition	Ankerbeispiel	Codierregel
K1: Bereiche der klinischen Patientenversorgung	Klinische Bereiche, die sich teilweise oder ganz mit der stationären Patientenversorgung beschäftigen.	Notaufnahme, Intensivstation, IMC, Normalstation, Operationszentrum, Pflegeeinrichtung	Es wird mindestens ein Tätigkeitsbereich in der stationären Patientenversorgung beschrieben.
K2: Bereiche der Infektionsprävention	Medizinische Tätigkeitsbereiche, die sich mit infektiologischer Prävention und Diagnostik beschäftigen.	Impfzentrum, Teststation, Test-Labor, Infektiologie	Es wird mindestens ein Tätigkeitsbereich in Strukturen der Infektionsprävention (primäre oder sekundäre Prävention) beschrieben.
K3: Bereiche der ambulanten Patientenversorgung	Medizinische Tätigkeitsbereiche in der ambulanten oder außerklinischen Patientenversorgung	Ambulante medizinische Versorgung, Krankentransportdienst, Rettungsdienst	Es wird mindestens ein Tätigkeitsbereich in der ambulanten oder außerklinischen Patientenversorgung beschrieben.
K4: Klinische Funktionsbereiche	Medizinische Funktionsbereiche, die keinen Beitrag zur infektiologischen Prävention oder Diagnostik leisten.	Radiologie, Laboratoriumsmedizin	Es wird mindestens eine Tätigkeit in einem klinischen Funktionsbereich beschrieben. Ausgenommen sind Bereiche, die unter Kategorie K2 einzuordnen sind.
K5: nicht-klinische Tätigkeitsbereiche	Tätigkeitsbereiche, die keinen Beitrag zur medizinischen Versorgung von Patienten beitragen.	Innerklinischer Patiententransportdienst, Gesundheitsamt, innerklinischer Sicherheitsdienst	Es wird mindestens eine nicht-klinische Tätigkeit beschrieben, die nicht unter den o.g. Kategorien fällt.

IMC: Intermediate Care Station

Welche Vorbereitung und Unterstützung bräuchte Ihre Abteilung / Ihr Bereich, damit der Einsatz zusätzlicher studentischer Hilfskräfte möglichst reibungslos ablaufen kann?

Kategorie	Definition	Ankerbeispiel	Codierregel
K1: Fachliche Sicherstellung der Einsatzfähigkeit	Für den reibungslosen Einsatz sollte die fachliche Einsatzfähigkeit sichergestellt werden. Hierfür sollten bspw. Einarbeitungskonzepte etabliert sein, Vorqualifikationen der Hilfskräfte berücksichtigt werden, Ressourcen zur Einarbeitung bereitgestellt werden und eine ausreichende Supervision während des Einsatzes sichergestellt sein.	"Einarbeitung auf der entsprechenden Station"	Die Antwort weist auf die Notwendigkeit einer fachlicher Sicherstellung der Einsatzfähigkeit hin.
K2: Adäquate Personalverwaltung	Administrative und verwaltungstechnische Hürden sollte ausreichend überwunden sein. Hierzu gehören bspw. die Beschaffung von betrieblichen Arbeitsmittel, EDV-Zugangsbereichtigungen, Schutzausrüstung und den Umgang mit den akademischen Pflichten der Studierenden.	"Komplette Administative Vorbereitung der Hilfskräfte: Berufskleidung Transponder Zugang zu PDMS, LAMP, Labor"; "Anerkennung der Tätigkeit"	Die Antwort weist auf die Notwendigkeit einer ausführlichen Personalverwaltung zur Sicherstellung der Einsatzfähigkeit hin.
K3: Klare strategische und operative Rahmenbedingungen	Die organisatorische Einbettung der Hilfskräfte an ihrem Einsatzort sollte sichergestellt sein. Hierzu zählen bspw. die adäquate Personalplanung und Tätigkeitsplanung.	"Fixe Zuordnung einer Anzahl an Studierenden zu einzelnen Abteilungen"	Die Antwort weist auf die Notwendigkeit der Klärung strategischer und operativer Rahmenbedingungen hin.

Welche Vorteile sehen Sie im Einsatz ausgebildeter studentischer Hilfskräfte in der Bewältigung einer medizinischen Notlage in Ihrer Abteilung?

Kategorie	Definition	Ankerbeispiel	Codierregel
K1: Entlastung des Stammpersonals	Der Einsatz würde das Stammpersonal in ihrem Arbeitsalltag entlasten, bspw. durch Zuarbeit, Optimierung von Arbeitsabläufe und eigenständige Übernahme diverser Tätigkeiten.	"Support, spezialisiertes Personal kann sich auf Vorbehaltstätigkeiten konzentrieren"	Die Antwort weist auf Vorteile des Einsatzes von Studierenden durch die direkte Arbeitsentlastung des Stammpersonals hin.
K2: Strategische und operative Vorteile	Der Einsatz von Medizinstudierenden bringt Vorteile in strategische und operative Abläufe mit sich, die nicht durch die direkte Entlastung von Stammpersonal zu erklären ist, bspw. durch ihre personelle Verfügbarkeit oder durch das Vorhandensein von Vorkenntnissen.	"geringere Einarbeitungszeit und schnellere Verfügbarkeit durch Ausbildung der studentischen Hilfskräfte"	Die Antwort weist auf Vorteile des Einsatzes von Studierenden für die Personalplanung hin, die nicht unter Kategorie K1 einzuordnen sind.
K3: Vorteile für die Studierende	Der Einsatz von Medizinstudierenden als Hilfskräfte würde sich positiv auf die Studierenden auswirken.	"[...] die Studenten können nebenbei gecoacht werden, Lernen das Leben im KH kennen"	Die Antwort weist auf einen Vorteil für die Studierenden durch ihren Einsatz hin.
K4: Vorteile für Patienten & Angehörige	Der Einsatz würde sich positiv auf die Versorgungsqualität und die Betreuung von Patienten und Angehörigen auswirken.	"Übertragung von Aufgaben aus ärztlicher oder pflegerischer oder sozialen Bereiche führen zu Entlastung, Ansprechpartner für Patienten und Angehörige führen zu weniger Verlust an Orientierung"	Die Antwort weist auf Vorteile des Einsatzes von Studierenden für die Patientenversorgung hin.

Welche Limitation sehen Sie für einem solchen Einsatz?

Kategorie	Definition	Ankerbeispiel	Codierregel
K1: Strategische und operative Limitationen	Der Einsatz wäre durch Aspekte der operativen Planung am Einsatzort limitiert. Hierzu gehören bspw. Aspekte der Personalplanung, Delegierbarkeit von Aufgaben und die Einbettung in den Strukturen der Klinik.	"Übliche Grenzen der Delegation"; "[...] Rechtliche Einschränkungen. Dürfen nicht eigenverantwortlich arbeiten."; "Zeitliche Verfügbarkeit der Hilfskräfte im Rahmen derer sonstigen Verpflichtungen"	Die Antwort weist auf strategische oder operative Hürden hin, die den Einsatz der Studierende limitieren könnten.
K2: Fachliche Limitationen	Der Einsatz wäre durch das (mangelnde) fachliche Wissen oder mangelnde Einarbeitung der Studierenden limitiert.	"Fehlende Erfahrung im Umgang mit Intensivpatienten [...]"	Die Antwort weist auf fachliche Mängel bei studentischen Hilfskräften hin.
K3: Nachteile für die Studierenden	Der Einsatz würde sich negativ auf die Studierende auswirken.	"[...] billige Arbeitskräfte werden ausgenutzt"	Die Antwort weist auf eine nachteilige Auswirkung des Einsatzes auf Studierenden hin.
