# Supplementary material for: Medical students in hospital emergency preparedness in the wake of the COVID-19 pandemic: a qualitative analysis of the ESCAPE study
Source: Bundesgesundheitsblatt Gesundheitsforschung Gesundheitsschutz. 2026 May 4;69(6):666–75. [Article in German] doi: 10.1007/s00103-026-04236-4 (PMC13212819; doi:10.1007/s00103-026-04236-4)
Supplement: Supplementary file 3 — ESM 3 Studentische Teilnehmer nach Fakultät [file 103_2026_4236_MOESM3_ESM.pdf]

<u>Onlinematerial 3</u>: Studentische Teilnehmer der Studierendenumfrage nach Fakultät

Onlinematerial 3: Studentische Teilnehmer der Studierendenumfrage nach Fakultät.

Medizinische Fakultät	Studentische Teilnehmer
RWTH Aachen University	145
Universität Augsburg	39
Ruhr-Universität Bochum	10
Universität Duisburg-Essen	97
Universität Düsseldorf	1
Goethe-Universität Frankfurt	1
Georg-August-Universität Göttingen	167
Universität Greifswald	5
Universität Hamburg	31
Medizinische Hochschule Hannover	139
Ruprecht-Karls-Universität Heidelberg - Medizinische Fakultät Mannheim	1
Christian-Albrechts-Universität zu Kiel	104
Universität zu Lübeck	90
Philipps-Universität Marburg	9
Ludwig-Maximilians-Universität München	112
Technische Universität München	116
Carl von Ossietzky Universität Oldenburg	1
Universität Regensburg	43
Universität Rostock	131
Universität des Saarlandes	10

Sieben Studierende hatten zwei Fakultäten angegeben, 3 Studierende hatten keine Aussage zur Fakultät gemacht.
